# Knowledge, Attitude, and Barriers Towards Human Papillomavirus (HPV) Vaccination Among Youths of Karachi, Pakistan

**DOI:** 10.7759/cureus.6134

**Published:** 2019-11-12

**Authors:** Mahnoor Y Shaikh, Maheen F Hussaini, Mehek Narmeen, Rida Effendi, Neha S Paryani, Ameer Ahmed, Muhammad Khan, Hasan Obaid

**Affiliations:** 1 Internal Medicine, Dow University of Health Sciences (DUHS), Karachi, PAK; 2 Internal Medicine, Dow University of Health Sciences (DUHS), Karachi , PAK

**Keywords:** human papilloma virus (hpv), knowledge, attitude, barriers, vaccine, youth, karachi, pakistan

## Abstract

Introduction

Human papillomavirus (HPV) is the most common infection of the reproductive tract. The introduction of HPV vaccines by WHO aims to reduce the incidence of cervical cancer. Pakistan lacks an effective nationwide HPV vaccination program, thereby making HPV a major threat. In this study, we aimed to assess the knowledge of HPV vaccination in Pakistan and to determine the barriers against it.

Methods

A cross-sectional study was conducted in Karachi, Pakistan, between April and May 2019. A convenience sampling technique was implemented using a self-administered questionnaire, which was filled by individuals aged 18-26. The questionnaire assessed the knowledge regarding HPV and also evaluated the attitude and acceptability amongst these individuals towards the vaccine. Data were analyzed using Statistical Package for Social Sciences (SPSS), version 20.0.

Results

The majority of the participants belonged to the monthly household income range of 200,000 rupees and above, which was labeled as the high-income category (n=158, 39.5%). Out of the 18 people who were vaccinated, eight belonged to the aforementioned category. Of these 18, nearly two-thirds (n=11, 61.1%) had gotten vaccinated upon the recommendation of their doctor. A statistically significant difference was found only between those currently enrolled in universities and previously vaccinated for HPV (p=.047). Nearly half of the responders perceived the vaccine to be time-consuming (n=167, 41.8%) and overpriced (n=187, 46.8%).

Conclusion

The vaccination rate is low in Karachi, Pakistan. Concentrated efforts involving the healthcare system should be made to raise awareness regarding HPV and its vaccine thereby reducing barriers to HPV prevention

## Introduction

Human papillomavirus (HPV) is the most common infection of the reproductive tract [1]. This virus can be sorted into two subtypes, high-risk and low-risk. Low-risk types such as HPV 6 and 11 cause lesions in the genitalia but are not deemed carcinogenic. On the other hand, high-risk types such as HPV 16 and 18 are responsible for about 75-80 percent of cervical cancers worldwide [2,3]. About 84.3% of all cervical cancers were reported from developing countries alone; out of cervical cancer patients in Pakistan, 88.3% of women had HPV type 16 or 18 or both. Today, more than 60 million females aged 15 or above are at risk of cervical cancer, with a crude incidence rate of 5.97 per 100,000 [4,5].

The HPV vaccine has been recommended by WHO as the prime approach for the prevention of cervical cancer, and should ideally be administered prior to first sexual contact [6]. In 2006 and 2007, two HPV vaccines, Gardasil® (Merck & Co., Whitehouse Station, NJ, USA) and Cervarix® (GlaxoSmithKline Biologicals, Rixensart, Belgium) were approved in the United States of America and Europe, respectively, and have since been licensed in more than 100 countries worldwide including Pakistan [7]. The introduction of HPV vaccination aims to reduce the incidence of cervical cancer [8].

Being a lower-middle-income country with a high burden of cervical cancer, Pakistan lacks an effective nationwide HPV screening and vaccination program. As a result, the Pakistani population may be unaware of there being such a vaccine. The potential of a vaccine to reduce disease burden relies on its acceptance and uptake by the community. Effective community health education and awareness-raising is a key component of any vaccination program [9].

Furthermore, there have been very few studies related to HPV vaccination in developing countries as compared to developed countries worldwide [10,11]. Thus, a study regarding knowledge, attitude and barriers towards HPV vaccination could not only help health-care planners to successfully formulate the most efficient plan for the prevention of HPV infections in Pakistan but also narrow the knowledge gap related to HPV vaccination between developed and developing countries. Considering the paucity of data, especially in our part of the world, the primary objective of this study was to assess the knowledge of HPV vaccination amongst individuals in Pakistan. The secondary objective of this study was to determine the barriers that prevent the uptake of the HPV vaccine in Pakistan.

## Materials and methods

A cross-sectional study was conducted in Karachi, Pakistan, between April and May 2019. The sample size was calculated using openepi.com. Considering the population size of 1,000,000 with an anticipating frequency of 53%, a sample size of 383 was deduced at a 95% confidence interval. All individuals belonging to the age group of 18 to 26 years who could comprehend the questionnaire and of sound cognitive skills were included in the study, regardless of any other demographic limitation. A convenience sampling technique was implemented using a self-administered questionnaire. Certain methods were adopted to minimize bias, which included, but were not limited to, convenience sampling by each author covering different areas of Karachi and including participants from various socioeconomic backgrounds. Additionally, the questionnaire was peer-reviewed, and a pilot study was conducted.

Following an extensive literature review, a standardized questionnaire was designed and distributed among participants. The self-administered questionnaire provided careful instructions and a brief description of the motives behind the study. Informed consent was obtained from each respondent, with the choice of anonymity due to certain personal demographics being inquired. The questionnaire was designed to not only inculcate knowledge regarding HPV but also to evaluate the attitude and acceptability amongst these individuals towards the vaccine. It was divided into three main parts, which incorporated socio-demographics, knowledge, and attitude. The first part included details regarding age, sex, relationship status, household income, the field of study, and employment status. The second section addressed the pre-existing knowledge about HPV, its transmission, associated diseases, and certain personal attributes, including sexual activity, past vaccinations, and if female, Pap smear status. Thirdly, there was an inquiry regarding previously received HPV vaccination and the respondents' own opinions about its application, including availability, presumed side effects, and cost.

Data were analyzed using Statistical Package for Social Sciences (SPSS), version 20.0. Categorical variables were expressed using frequencies and percentages. Continuous variables were presented as a mean and standard deviation. A chi-squared test with a 95% confidence interval was used to compare categorical variables.

## Results

**Figure 1:**
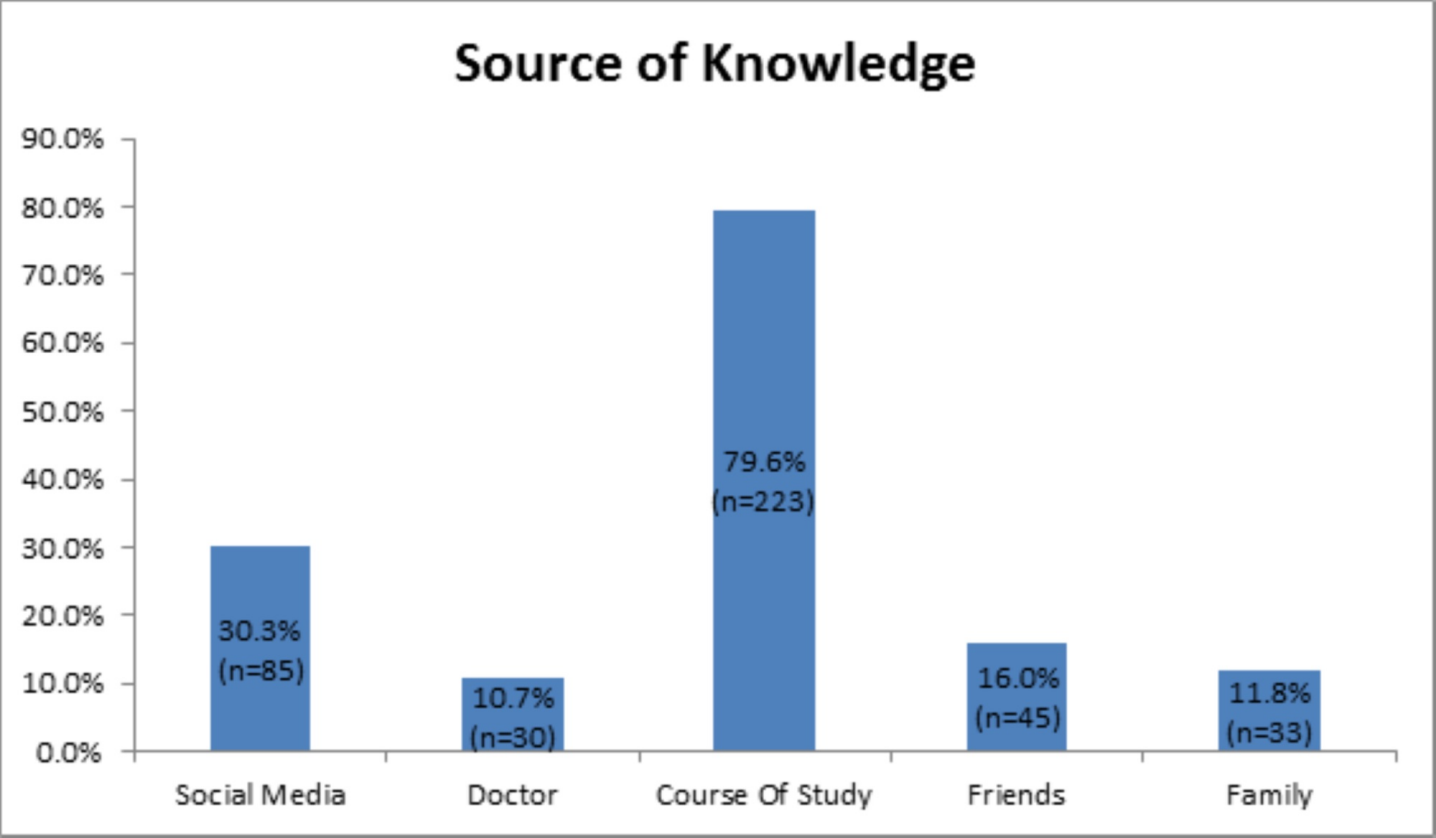
Source of Knowledge

A total of 400 people participated in this study, out of which nearly three quarters were female (n=280, 70.0%) (Table 1). The mean age of respondents was 22.1 ± 2.1 years, whereas the mean age at which participants were vaccinated for HPV was 15.3 ± 4.7 years. The participants were mostly single (n=337, 84.3%), unemployed (n=300, 75.0%), university students (n=270, 67.5%) and/or associated with the medical field (n=242, 60.5%). An overwhelming proportion of the participants (n=223, 79.6%) cited the course of study as their source of knowledge (Figure 1). The majority of the participants belonged to the monthly household income range of 200,000 rupees and above, which was labeled as the high-income category (n=158, 39.5%). Only 18 of the 400 (4.5%) subjects that took part in this study were previously vaccinated for HPV, 8 of whom belonged to the aforementioned socio-economic category. Of these 18, nearly two-thirds (n=11, 61.1%) had gotten vaccinated upon the recommendation of their doctor. Furthermore, only about a quarter of all our respondents were sexually active (n=95, 23.8%). Out of the social demographic categories, a statistically significant difference was found only between those currently enrolled in universities and previously vaccinated for HPV (p=.047).

**Table 1:** Sociodemographics of Participants

Characteristics	n (%)	Previously vaccinated For HPV (%)	P-value
Gender			.207
Male	120 (30.0)	3 (2.5)	
Female	280 (70.0)	15 (5.4)	
Employment Status			.781
Employed	100 (25.0)	4 (4.0)	
Unemployed	300 (75.0)	14 (4.7)	
Relationship Status			.561
Single	337 (84.3)	15 (4.5)	
Engaged	39 (9.8)	3 (7.7)	
Married	21 (5.3)	0 (0.0)	
Divorce	3 (0.8)	0 (0.0)	
Currently Enrolled in a University			.047*
Yes	270 (67.5)	16 (5.9)	
No	130 (32.5)	2 (1.5)	
Field Of Study			.584
Medicine	242 (60.5)	12 (4.9)	
Others	158 (39.5)	6 (3.8)	
Household Income (Pakistani Rupees)			.245
<50,000	60 (15.0)	2 (3.3)	
50,000-100,000	72 (18.0)	1 (1.4)	
100,000-150,000	51 (12.8)	5 (9.8)	
150,000-200,000	59 (14.8)	2 (3.4)	
>200,000	158(39.5)	8 (5.1)	
Sexually Active			.122
Yes	95(23.8)	7(7.4)	
No	305(76.2)	11(3.6)	

When asked about various aspects of HPV, more than 2/3rd of the participants responded with the correct answers, with 91% (n=364) of the participants correctly recognizing HPV’s ability to infect both males and females (Table 2). Close to a quarter (n=73) of the participants did not consider sexual contact as a route of transmission, and more than a quarter of the respondents believed that HPV has no association with cervical cancer (n=109, 27.3%). Additionally, respondents believed that the HPV vaccine protected against breast cancer (n=32, 8%), anal cancer (n=89, 22.3%), vulvar cancer (n=124, 31%) and HIV (n=77, 19.3%). When questioned about the recommended age for vaccination, only 33% (n=132) knew the correct age for vaccination as being 9-14 years, out of which 65.9% (n=87) respondents belonged to the medical background. From a total of 95 sexually active individuals in the study, 51 were females (Table 1). Amongst them, only five (9.80%) were previously screened for cervical cancer via Pap smear.

**Table 2:** Prior Knowledge regarding HPV

Knowledge Assessment Questions	n (%)
What does HPV stand for?	
Human Papillomavirus	351 (87.8)
Other	49 (12.3)
How is HPV transmitted?	
Contaminated Food	26 (6.5)
Sneezing/Coughing	27 (6.8)
Physical Contact	20 (5.0)
Sexual Contact	327 (81.8)
Who does HPV infect?	
Male	2 (0.5)
Female	34 (8.5)
Both	364 (91.0)
Does HPV always have symptoms?	
Yes	115 (28.7)
No	285 (71.3)
Is HPV associated with cervical cancer?	
Yes	291 (72.8)
No	109 (27.3)
Is HPV associated with Genital Warts?	
Yes	304 (76.0)
No	96 (24.0)

**Figure 2:**
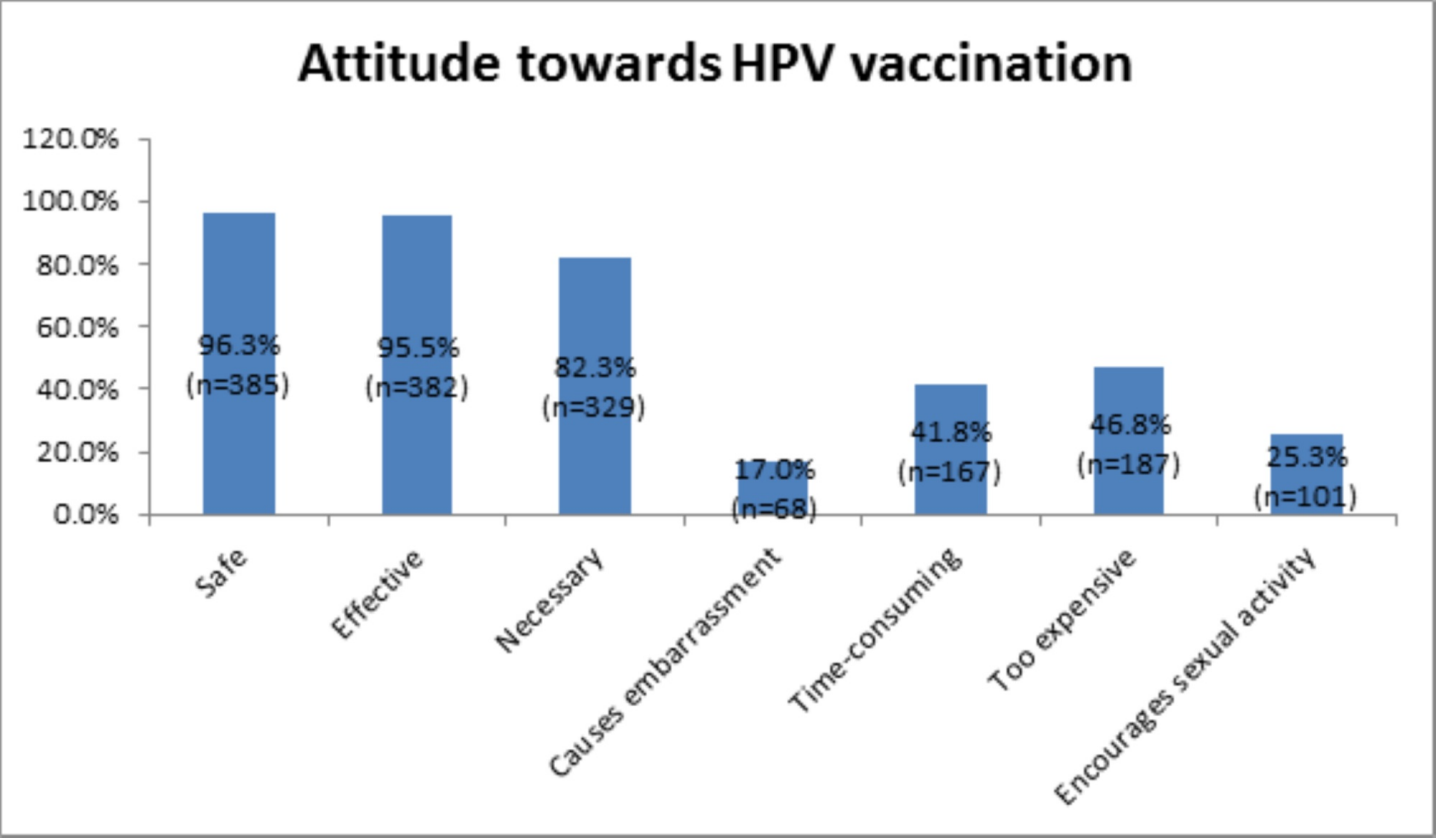
Attitude towards HPV vaccination

Figure 2 illustrates that the majority of participants considered the HPV vaccine to be safe, effective, and necessary. However, a quarter of the study population (n=101) believed that HPV vaccination promoted sexual activity in young adults. Nearly half of the responders perceived the vaccine to be time-consuming (n=167, 41.8%) and overpriced (n=187, 46.8%).

Out of the participants that were not vaccinated for HPV (n=382, 95.5%), the intention for a future HPV vaccine is seen in 62.0% of subjects (n=237). The resolve for a future HPV vaccination is higher in females (n=185, 78.1%). There is a statistically significant difference between the intention to achieve HPV immunization in the future and gender, with females more likely to get vaccinated (p<0.001). Moreover, from the population unvaccinated for HPV, 95.5% (n=365) claimed that they would consider getting vaccinated for HPV in the future if their doctors recommended it.

## Discussion

Interestingly, the majority of the participants in our study had sufficient knowledge of HPV. This is in contrast to a similar study conducted by Zhuang et al. among female students studying in a tertiary institute in Singapore wherein upon administering a 14 point grading system to assess knowledge of HPV it was seen that the participants had insufficient knowledge with a median score of 7 [12]. As expected, medical students were significantly better informed about HPV and its associated vaccine due to their course of study; however, the non-medical students were not as well-aware, as is concurrent with other studies [13,14]. The course of study was cited as the most common source of knowledge amongst our participants, highlighting the role of schooling in raising awareness regarding pertinent diseases such as HPV. This can also be supported by studies showing that better education regarding HPV leads to an increased willingness to get vaccinated [15].

As predicted, females were more inclined to vaccinate themselves against HPV than men [16]. The reason for this could be two-fold. Firstly, cervical cancer is statistically proven to be the most devastating possible outcome of HPV, and it is only women who are affected by it. This, coupled with the fact that awareness regarding the vaccine's importance is already extremely limited, helps to understand why HPV vaccination and screening programs tend to target the female population more, if not exclusively. Hence, the increased propensity for women towards future HPV vaccinations is a reflection of them being more knowledgeable about the matter [17]. Not surprisingly, the people who were already vaccinated against HPV had done so on the recommendation of the doctor, signifying the crucial role health care professionals play in raising awareness and promoting the acceptability of the HPV vaccine [18]. However, the number of vaccinated subjects in our study population was considerably low; this depicts the need for more effective and widespread communication between the physicians and the general population, to ensure that accurate information is dispelled and there is a rise in the uptake of HPV vaccine [19,20].

Although a notable number of participants were convinced regarding the safety, necessity, and efficacy of the HPV vaccine, thus eliminating the need to emphasize on vaccine safety, nearly half of them claimed that they did not have the time to get vaccinated and that the vaccine was expensive [21]. A study carried out amongst health sciences students in Malaysia by Rajiah et al., exhibited similar results wherein the participants reported the cost of the HPV vaccine as the biggest hindrance in obtaining HPV vaccination [22]. Furthermore, our study shows that the largest number of vaccinations belonged to the highest income group, hence establishing the relationship between income and the likelihood of vaccination, mirroring the findings of Islam et al. [23]. This correlation is transparent in an underdeveloped country such as Pakistan, where most people are focused on being able to provide the basic necessities for their families. This, coupled with the fact that quality healthcare is not within easy reach for the entire population, save for a select few, often translates into health not being a top priority for most people. Under such circumstances, the government should offer free or subsidized vaccinations that are readily available to the general population, specifically young adolescents; the recommended age for vaccination is 9-14 years, as studies have shown that the antibody titer is highest against all HPV genotypes if vaccinated within this age group [24,25].

There is a stigma associated with HPV due to its association with cervical cancer, leading to a lack of open dialogue surrounding reproductive health in low and middle-income countries, making it difficult and sometimes nearly impossible to overcome preventable issues concerning sexual health [26]. There is a need for widespread, culturally sensitive educational campaigns involving family, especially parents; it is crucial to remove the preconceived notion and fear that the use of the HPV vaccine can lead to sexual promiscuity to break the stigma that is associated with the use of HPV vaccine [27]. In addition, since ours is a traditional set-up, the autonomy for making such decisions often lies in the hands of the parents; it is imperative for them to be well-educated about this topic so that their children can get vaccinated, and possibly reduce the risk of developing HPV-associated malignancies [28]. Furthermore, prevention and screening programs should also target males as most males are unaware of the fact that HPV can cause numerous diseases in men too, including genital warts, penile and anal cancer [17]. In addition, other mediums such as television and social media should be used to raise awareness regarding the safety and effectiveness of the HPV vaccine, thereby encouraging a positive attitude towards it. Since this pilot study was conducted only in Karachi, a similar study conducted on a larger scale encompassing multiple rural and urban areas of the country would provide a nationwide result on the knowledge, acceptability, and uptake of the HPV vaccine. A countrywide immunization campaign targeting both men and women can then be initiated by the government, involving physicians who can dispel any of the false information associated with the HPV vaccine. 

Like all other studies, our study also has its limitations. Firstly, our sample size is not representative of the entire population as we used convenience sampling. Secondly, since this is a cross-sectional study, there is a risk of recall bias regarding certain questions, such as the sources of HPV-related information. Thirdly, there is also a risk of social desirability bias because this study was conducted on a sensitive topic, despite the participants having the option of filling out the questionnaire anonymously. Lastly, the sample size only consists of 400 students and can thus, reduce the generalizability of the results.

## Conclusions

According to our study, a significant portion of the population, especially females, were aware of the HPV vaccine and were inclined to get vaccinated as well. The men, on the other hand, were not as well-informed. However, the rate of vaccination uptake, for both genders, was not as high due to the exorbitant price, inaccessibility of the vaccine as well as the associated misconceptions that the use of the vaccine will encourage sexual promiscuity. Therefore, it is imperative that there is widespread awareness regarding the use of the vaccine targeted towards both genders. In addition, the vaccine should be readily available to all those who need it.
